# Changes in Corneal Densitometry and Relationship to Corneal Topographical Parameters in Accelerated Corneal Crosslinking for the Treatment of Keratoconus

**DOI:** 10.3390/jcm15083137

**Published:** 2026-04-20

**Authors:** Yifan Du, Hanyu Jiang, Fei Mo, Ying Li, Yang Jiang

**Affiliations:** 1 Department of Ophthalmology, Beijing Chaoyang Hospital, Capital Medical University, Beijing 100020, China; duyifan260930@163.com; 2 Department of Ophthalmology, Peking Union Medical College Hospital, Chinese Academy of Medical Sciences & Peking Union Medical College, Beijing 100730, China; 3 Key Laboratory of Ocular Fundus Diseases, Chinese Academy of Medical Sciences & Peking Union Medical College, Beijing 100730, China

**Keywords:** keratoconus, corneal collagen crosslinking, corneal densitometry, corneal topography

## Abstract

**Background/Objectives**: To evaluate changes in corneal topography and densitometry (CD) 3 months after accelerated corneal collagen crosslinking (CXL) for keratoconus and to investigate influencing factors. **Methods**: Twenty-one (41 eyes) patients with KC who underwent accelerated epithelium-off CXL were included in this retrospective observational study; preoperative and 3-month postoperative CD and corneal topographic parameters measured by Pentacam HR were collected. The changes in corneal topographic parameters and CD before and after CXL were subsequently compared, and the correlation between age, corneal topographic parameters and CD changes was analysed. **Results**: Except for TCT (*p* = 0.026), no significant changes were observed in topographic parameters (*p* > 0.05). There was a significant increase in total CD (tCD), CD 0–2 mm and CD 2–6 mm from the whole, anterior and central corneal layers after CXL compared to pre-operation (*p* < 0.05). The results of the correlating factors showed that age was positively associated with changes in tCD, CD 0–2 mm, and CD 2–6 mm from the whole, anterior and central corneal layer (*p* < 0.05), which was fully consistent with the regions treated by CXL. Other factors showed only marginal associations and were not consistent with the regions treated by CXL. **Conclusions**: An increase in CD can be observed in patients with KC at 3 months after undergoing accelerated CXL, and this is particularly focused on the 0–2 mm and 2–6 mm regions of the anterior and central corneal layers. The degree of change in CD was influenced by age, which may suggest differences in corneal response to CXL in KC of different ages.

## 1. Introduction

Keratoconus (KC) is a progressive non-inflammatory corneal degenerative disease that can lead to progressive thinning of the cornea, myopia, irregular astigmatism, and decreased visual acuity, which seriously affects the corneal health and quality of life [1,2]. The histopathological manifestations of KC are progressive degeneration of the corneal basal epithelial layer, followed by collagen fragmentation of the arcuate layer. The cornea becomes progressively thinner during the course of KC, with misaligned collagen layers [3]. Currently, corneal collagen crosslinking (CXL) is used as an effective treatment for progressive KC, which increases the corneal stiffness and reduces its compliance, which in turn is able to stop the progression of KC [4,5,6]. It uses ultraviolet light at a wavelength of 360–380 nm to irradiate the photosensitising agent riboflavin to induce crosslinking of collagen fibres within the corneal stroma, thereby enhancing the stability of the corneal stroma. Meanwhile, since the accelerated protocol was introduced in 2010, advantages such as shorter procedure times and lower complication rates have been achieved [7]. However, the quantitative evaluation of the effect of CXL for KC and the change in haze has become a problem that needs to be solved.

The emergence of corneal densitometry (CD) provides a direction to solve this problem. It is an inherent property of the cornea, correlates with the degree of corneal transparency, and can be used as an important index for evaluating corneal transparency [8]. Meanwhile, the emergence and commercial application of the Pentacam 3D preocular segment analysis system (Oculus, Germany) has made non-invasive and reproducible CD determination possible [8,9,10]. CD has now been used in KC, infectious keratitis, and corneal stromal dystrophy [10,11,12]. In contrast, there are a few studies with inconsistent conclusions on the application of CD to assess CXL for KC, and there is a lack of studies analysing the factors that influence the effectiveness of CXL [13,14]. The aim of this study was to investigate the changes in corneal topographic parameters and CD values in KC before and after undergoing accelerated CXL and to further investigate the association between these factors and the changes in CD, in order to enable the postoperative recovery assessment of KC undergoing CXL to provide a useful reference.

## 2. Materials and Methods

### 2.1. Subjects

This retrospective observational study collected past medical records of patients with progressive KC who visited Peking Union Medical College Hospital between 1 June 2023 and 1 June 2024; these patients underwent CXL as the primary sole surgical intervention. The patients underwent a thorough preoperative eye examination and were periodically re-examined after surgery. The inclusion criteria for this study were as follows: (1) subjects were required to be patients with progressive KC, defined as an increase in the maximum keratometry of at least 1.00 dioptre (D), an increase in the manifest cylinder of ≥1.00 D, or a manifest refraction spherical equivalent change of ≥0.5 D over the past 1 year; (2) bilateral minimum corneal thickness ≥ 380 μm; (3) the ages of these participants ranged from 15 to 50 years old; (4) subjects were not currently using topical ophthalmic medications; (5) subjects had not undergone any ocular surgery in the past and had no history of any other disease that may adversely affect the eye and vision; (6) no active inflammation. Preoperative evaluation included the following clinical history (age, complaints, ocular trauma or disease, any systemic disease, etc.) and relevant ophthalmological examination, slit lamp biomicroscopy and fundus examination. Corneal data including thinnest corneal thickness (TCT), Ks, Kf and relevant corneal aspheric parameters were collected from KC patients using corneal topography (Pentacam HR, Oculus, Wetzlar, Germany).

This study adhered to the principles outlined in the Declaration of Helsinki and was approved by the Institutional Review Board of the Peking Union Medical College Hospital (I-24PJ0096). Written informed consent was obtained after patients were identified for inclusion.

### 2.2. Corneal Densitometry Analysis

Patients with KC underwent examinations of CD preoperatively and 3 months postoperatively, and these data were obtained by rotational Scheimpflug camera imaging (Pentacam HR, Oculus, Wetzlar, Germany). Measurements were performed by an expert operator (YD) in a standard dimly lit room under the same conditions for all patients. Measurements were repeated three times for each patient, and only results with a quality check of “OK” were considered for analysis. After measurement, the CD was quantified in grey scale units (GSU) from 0 GSU (maximum value of corneal transparency) to 100 GSU (minimum value of corneal transparency). According to the depth-based anatomical corneal layers, the cornea was divided into four layers, including the superficial anterior layer of 120 μm, the innermost posterior corneal layer of 60 μm, the central layer where the thickness of the anterior and posterior layers was subtracted from the total layer thickness, and the total layer [15]. Each layer was subdivided into four concentric radial zones based on the distance to the corneal apex (0–2 mm, 2–6 mm, 6–10 mm and 10–12 mm) [16]. CD values for the 10–12 mm region were not included in this study because of poor reproducibility due to image boundary effects (strong reflections from the corneal limbus or sclera) [17].

### 2.3. Surgical Procedure

Accelerated epithelium-off CXL mode was used in all patients. Levofloxacin 0.5% eye drops (Santen Pharmaceutical Co., Ltd., Osaka, Japan) were instilled into the operative eye 4 times/d for 3 d. Oxybuprocaine 0.4% hydrochloride eye drops (Santen Pharmaceutical Co., Ltd., Japan) were used to spot the operative eye 3 times prior to surgery, and when surface anaesthesia was in effect, a 9 mm-diameter corneal ring drill was used to mark the eye along the central cornea, and the corneal epithelium was mechanically removed with a blunt spatula. Then, we applied a riboflavin salt solution (Vibex Rapid 0.1%, Avedro, Waltham, MA, USA), 1 time/2 min, for a total of 5 times for 10 min. After saline rinsing, a UV crosslinking reinforcement device (Avedro, USA) was applied, with a wavelength of 365 nm, an irradiance of 9 mW/cm^2^, and UV crosslinking irradiation for 10 min, with a total irradiation energy of 5.4 J/cm^2^. Antibiotic eye drops were placed in the eyes, and soft corneal contact lenses were worn in the operated eyes. After the operation, hormone and antibiotic eye drops were used to treat the eyes, and the corneal contact lenses were removed according to the healing of the corneal epithelium.

### 2.4. Statistical Analysis

Statistical analyses were performed using SPSS 25.0 (IBM, Chicago, IL, USA). Data normality was assessed using the Kolmogorov–Smirnov test, and paired Student’s *t*-tests and Wilcoxon signed-rank tests were used to compare normally and non-normally distributed corneal topography and CD data between the preoperative and 3-month postoperative time points. Spearman and Pearson correlation tests were used to assess associations between correlated factors and CD. Linear regression models were subsequently used to further explore the association between possible positive findings and CD. Continuous variables are expressed as mean ± standard deviation and categorical variables as percentage (%). Statistical significance was defined as a *p*-value less than 0.05.

## 3. Results

A total of 21 subjects (41 eyes), 12 males and 9 females, were included in this study. The mean age of these subjects was 26.05 ± 6.80 years, the mean Kf was 45.87 ± 3.53 D, the mean Ks was 48.99 ± 4.58 D, and the mean TCT was 468.00 ± 34.52 μm. All subjects underwent accelerated epithelium-off CXL. There were no significant differences in corneal topographic parameters at 3 months postoperatively (*p* > 0.05), except for the presence of a statistically significant reduction in TCT (*p* = 0.026). All patients showed no significant improvement in best-corrected visual acuity (BCVA) at 3 months postoperatively (logMAR visual acuity, pre-op: 0.32 ± 0.21 vs. post-op: 0.31 ± 0.23, *p* = 0.593). The specific results can be seen in Table 1.

**Figure 1:**
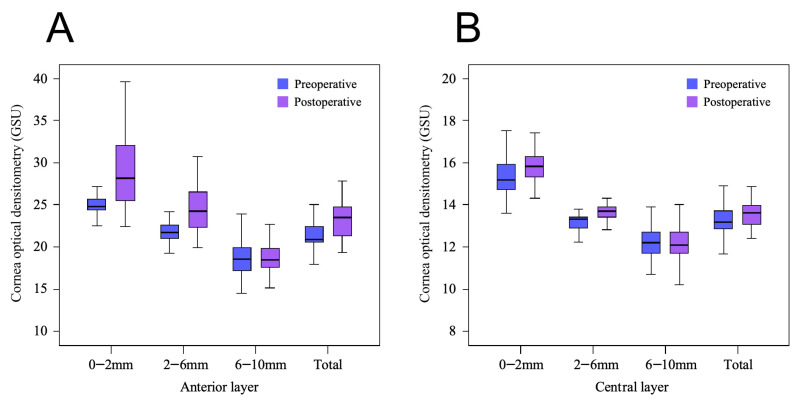
Pre- and postoperative CD values for each region of the anterior corneal layers (**A**) and central corneal layers (**B**) It can be observed that, in the anterior and central layers of the cornea, the 0–2 mm and 2–6 mm areas are the main regions where CD increases, while the 6–10 mm corneal area shows no significant change. Overall, an increase in CD can still be observed, with the anterior layer appearing more pronounced than the central layer.

**Figure 2:**
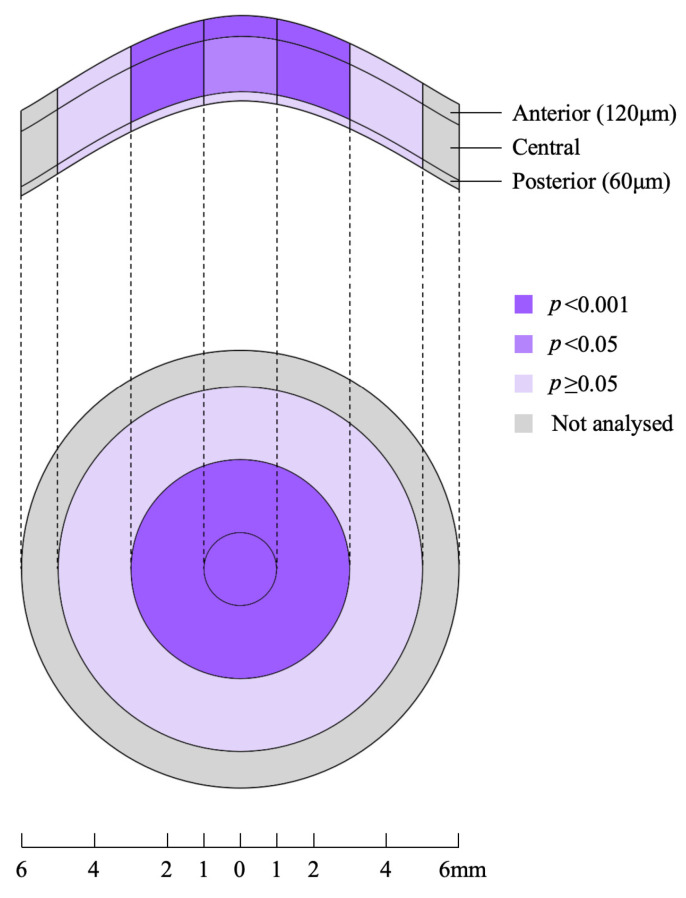
Visualisation of CD changes before and after CXL Through this figure, it can be visually observed that significant CD changes occur in the 0–6 mm area of the anterior and central layers of the cornea, while the changes in the remaining peripheral and posterior layers of the cornea are not obvious.

The preoperative findings showed a gradual decrease in CD from the anterior to posterior layers and from the central to periphery of the cornea, and this trend was maintained 3 months after CXL was performed. The comparison results showed that the whole total CD (tCD), whole 0–2 mm CD, whole 2–6 mm CD, anterior tCD, anterior 0–2 mm CD, anterior 2–6 mm CD, central tCD, central 0–2 mm CD, and central 2–6 mm CD increased significantly after CXL compared with the preoperative data (*p* < 0.05), whereas the 6–10 mm CD of each layer and all of the posterior layer CD were not changed significantly before and after CXL (*p* > 0.05); the specific CD changes are shown in Table 2 and Figure 1 and Figure 2.

**Figure 3:**
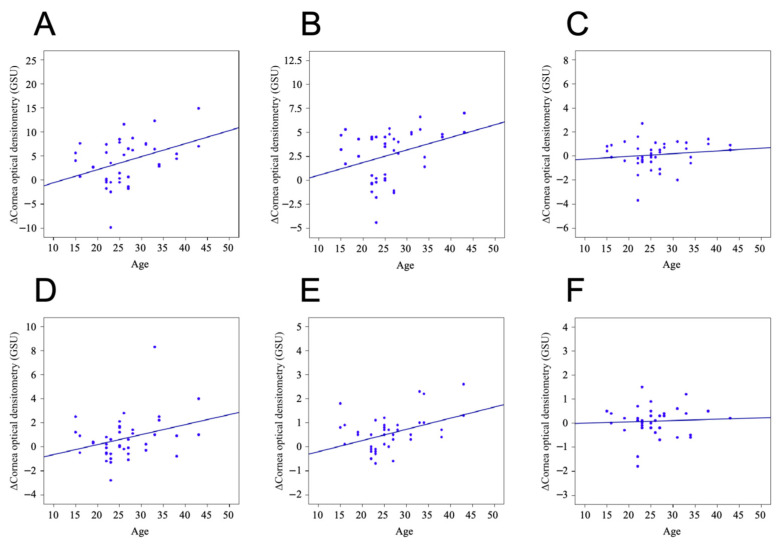
Association between age and CD changes: (**A**) anterior 0–2 mm; (**B**) anterior 2–6 mm; (**C**) anterior 6–10 mm; (**D**) central 0–2 mm; (**E**) central 2–6 mm; (**F**) central 6–10 mm. It can be seen that the difference in CD values in the anterior 0–2 mm, anterior 2–6 mm, central 0–2 mm, and central 2–6 mm of cornea gradually increases with age. That is, the older the patient, the higher the increase in CD after CXL. However, this trend is less pronounced in the peripheral cornea.

From the findings of the correlation factors, age was positively associated with changes in whole tCD (r = 0.377, *p* = 0.015), whole 0–2 mm CD (r = 0.397, *p* = 0.010), and whole 2–6 mm CD (r = 0.374, *p* = 0.016), whereas corneal topography factors were only positively associated with changes in Astig F, whole tCD (r = 0.341, *p* = 0.029) and whole 2–6 mm CD (r = 0.361, *p* = 0.021). The remaining factors were not significantly associated with CD changes in each region (*p* > 0.05). Specific results can be seen in Table 3. For the analysis of each layer, this study found a pattern of positive associations with changes in CD only for age, which was consistent with the regions of significant CD altered by CXL (anterior layer and central layer 0–2 mm and 2–6 mm), whereas the other factors were only sporadically associated with changes in CD. This association is shown in Figure 3. Specific results can be seen in Supplementary Table S1.

## 4. Discussion

**Figure 4:**
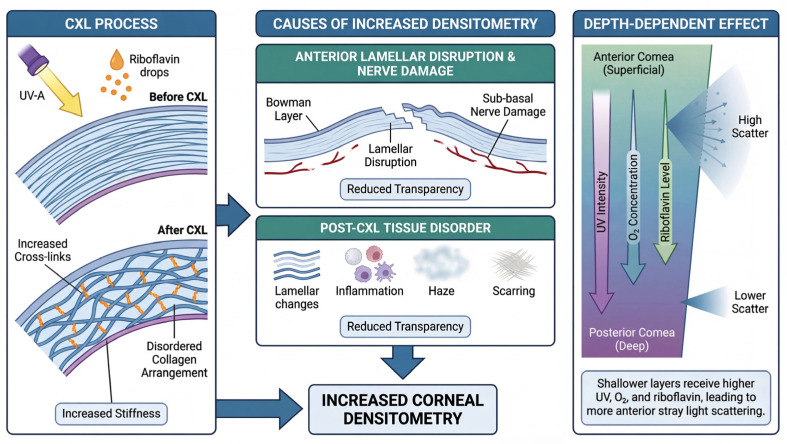
Mechanisms of increased CD after CXL.

Our results showed a significant increase in CD in the anterior and central corneal layers after accelerated epithelium-off CXL, which was most pronounced in the two central concentric zones (0–2 mm and 2–6 mm). This is consistent with previous reports by Böhm et al. [18], who observed a significant increase in CD after accelerated CXL, which was most evident in the region of the 0–2 mm anterior corneal layer. In contrast, Mohebbi et al. [19] found that an increase in CD is frequently observed in the first months after CXL, which corresponds to the haze detected clinically during that period. Pircher et al. [20] showed that standard CXL induces various changes in the corneal stroma, which leads to an increase in CD, especially in the anterior corneal layer and the 0–2 mm region of the central corneal layer. Therefore, it can be hypothesised that light scattering is more prominent in the anterior corneal layer after CXL due to UV light intensity, decreasing oxygen concentration towards the middle and deeper stromal layers, and riboflavin concentration. However, it has been suggested that since the main effect of CXL is to increase the interconnections between the corneal stromal collagen to achieve increased corneal stiffness [21], this can lead to a disruption in the alignment of the corneal stromal fibres, which in turn causes an increase in corneal scattering, leading to an increase in CD [21,22]. Therefore, the main reason for the increase in CD is the rupture of Bowman’s layer and the damage of the sub-stromal nerve fibres, which leads to a decrease in corneal transparency and an increase in CD [23]. Corneal histological disturbances (e.g., changes in corneal lamellar alignment and spacing, inflammation, haze, and scarring) may affect corneal transparency and are considered the most likely cause of increased CD [11,24]. Figure 4 illustrates the mechanisms by which the aforementioned CD increases after CXL.

For the trend of CD after CXL surgery, this study found a significant increase in CD at 3 months, whereas some studies have regularly monitored the change in CD values after CXL. Alzahrani et al. [25] examined the CD values of KC before CXL, 3 months after CXL, 6 months after CXL, and 1 year after CXL; the results showed that the CD values of the 0–2 mm and 2–6 mm corneal region were the highest at 3 months after CXL, and they were significantly different from those of the preoperative and 6 months after CXL. The studies of Pircher et al. and Kim et al. [20,26] also suggested that CD gradually increased and reached a maximum after CXL and recovered at 12 months postoperatively, whereas there was a difference between these two studies in the time at which CD appeared to be at its peak, which was 3 months for the former and 1 month for the latter. This may be due to the fact that the exact timing of the appearance of the highest values of CD is not the same, due to differences in follow-up and specific surgical protocols. So, the timing of the peak appearance of CD after CXL still needs further experimental exploration. Xia et al. [27] observed New Zealand white rabbits after CXL using polarised light microscopy, and the results suggested that type 3 collagen increased significantly at 1 month compared with the control group, reached a maximum at 3 months, and was comparable to the control group’s level at 6 months postoperatively, which suggests that the synthesis and degradation of type 3 collagen disrupts the orderly arrangement of corneal collagen, thereby increasing light scattering and thus increasing CD.

After 3-month follow-up, this study found that patients with KC did not show significant changes in corneal topographic parameters other than TCT after CXL, while only some of these preoperative corneal topographic parameters (Astig F, TCT, Ecc F, Aspher F, and Kmax) correlated with the changes in CD and did not coincide with the treatment area of CXL. The changes in TCT are consistent with recent findings that TCT decreases after CXL [28,29], although the physiological cause of corneal thinning after CXL remains undetermined. However, changes in other corneal parameters have been observed in a number of studies. Nicula et al. [30] found a 0.6 D decrease in the mean Kmax 6 months postoperatively in KC patients undergoing CXL and an even more pronounced decrease in the mean Kmax 10 years postoperatively. Jabbarvand et al. [31] found that K1 and K2 on the corneal front surface were also significantly lower 6 months after CXL compared to 1 month after CXL. It was considered that this may be due to normal postoperative corneal stress response and corneal remodelling, although these changes were not observed in this study.

The present study also identified age as a key factor influencing the change in CD after CXL, which was demonstrated by the fact that with increased age, there will be a more pronounced increase in CD after CXL compared to the preoperative presence. In the corneal regions, there is a close relationship between age and CD change, coinciding with the main treatment areas of CXL, which has not been suggested in previous studies. However, age has been generally recognised as one of the parameters affecting CD. Sorcha et al. found in healthy Caucasians that CD increased with age. [17] Garzón et al. [32] also found that CD changes in all layers of the cornea were also positively correlated with age in healthy participants from Spain. However, Wei et al. [33] noted that there was no statistical correlation between CD and age in Chinese myopic patients. However, it is reasonable to believe that with increased age, degenerative changes in the corneal tissue and decreased water content can lead to a decrease in corneal clarity, resulting in an increase in CD. The phenomenon of increased CD after CXL with age may indicate a more pronounced disturbance of corneal histology or an increase in corneal stiffness, but longer follow-up will allow a better analysis of this process of change. At present, it suggests differences in outcome and healing when CXL is performed at different ages.

This study still has some limitations. 1. The sample size of this study is small; so, the results need to be confirmed by studies with larger samples. 2. There may be a correlation between the two eyes of a patient, which is a common error in ophthalmological studies, and thus may lead to a possible underestimation of the overall variance of the samples measured in both eyes, which may increase the risk of a type 1 error [34]. 3. The follow-up was for a single time period (3 months); therefore, the results could only reflect the changes in early corneal conditions after CXL. So, more frequent and longer follow-up times would allow for better observation of the trends in CD and the long-lasting effects on CD after CXL.

## 5. Conclusions

In conclusion, accelerated CXL can cause an increase in CD during the treatment of patients with KC, which can be observed at least at the 3-month postoperative time point. In this case, the increase in CD was concentrated in the anterior and central layers of the cornea, especially in the 0–2 mm and 2–6 mm regions. In contrast, there was no significant change in CD in the posterior corneal layer and the 6–10 mm peripheral corneal region. Except for TCT, the other corneal topographic parameters did not show significant changes before and after CXL, and there was also no specific correlation with changes in CD. The association between age and changes in CD may be of interest and suggest an effect of age on the effectiveness of CXL in treating KC. Further studies in the future could focus on this finding, and the changes in CD over a longer period of time after CXL will also be a key area worthy of further research.

## Figures and Tables

**Table 1:** Changes in corneal topography parameters via corneal crosslinking of keratoconus.

Parameters	Preoperative	Postoperative	*p*
Kf (D)	45.87 ± 3.53	45.80 ± 3.72	0.539
Ks (D)	48.99 ± 4.58	48.89 ± 4.51	0.555
TCT (μm)	468.00 ± 34.52	464.44 ± 34.37	0.026 *
Astig F (D)	3.11 ± 2.12	3.10 ± 1.95	0.889
Ecc F	0.86 ± 0.24	0.84 ± 0.28	0.236
Aspher F	−0.84 ± 0.41	−0.83 ± 0.44	0.684
Ecc B	0.85 ± 0.30	0.86 ± 0.29	0.397
Aspher B	−0.86 ± 0.45	−0.87 ± 0.46	0.521
Kmax (D)	54.44 ± 7.26	54.14 ± 7.28	0.141
ISV	71.12 ± 35.76	72.12 ± 38.98	0.624
IVA	0.71 ± 0.40	0.71 ± 0.42	0.825
I-S	4.07 ± 2.85	4.15 ± 3.35	0.772
KISA% ^†^	1383.23 ± 3697.84	1322.26 ± 2982.47	0.758

Aspher B = back corneal asphericity; Aspher F = front corneal asphericity; Astig F = front corneal astigmatism; Ecc B = back corneal numeric eccentricity; Ecc F = front corneal numeric eccentricity; ISV = index of surface variance; IVA = index of vertical asymmetry; Kf = flat keratometry; Ks = steep keratometry; TCT = thinnest corneal thickness. Kmax refers to maximum anterior sagittal curvature; I-S refers to difference in refractive power between inferior and superior at 6 mm diameter of cornea; KISA% refers to values based on a combination of keratometry, I-S, skewed radial axis, and astigmatism in Pentacam. Values are displayed in the form of mean ± standard deviation. *: *p* values < 0.05 at *t*-test. ^†^: These data are strongly affected by outliers.

**Table 2:** Changes in corneal optical densitometry via corneal crosslinking of keratoconus.

CD		Preoperative	Postoperative	Difference	*p*
Anterior layer	0–2 mm	25.26 ± 2.14	29.04 ± 4.28	3.78 ± 4.79	<0.001 *
	2–6 mm	21.84 ± 1.37	24.49 ± 2.66	2.65 ± 2.66	<0.001 *
	6–10 mm	19.16 ± 3.40	19.26 ± 3.32	0.10 ± 1.12	0.570
	Total	21.45 ± 1.76	23.31 ± 2.56	1.86 ± 2.19	<0.001 *
Central layer	0–2 mm	15.36 ± 0.94	16.04 ± 1.65	0.68 ± 1.79	0.019 *
	2–6 mm	13.21 ± 0.56	13.75 ± 0.71	0.54 ± 0.78	<0.001 *
	6–10 mm	12.40 ± 1.56	12.48 ± 1.55	0.08 ± 0.60	0.396
	Total	13.32 ± 0.79	13.70 ± 0.99	0.38 ± 0.73	0.002 *
Posterior layer	0–2 mm	10.00 ± 0.88	10.10 ± 1.23	0.10 ± 1.14	0.567
	2–6 mm	10.31 ± 0.73	10.40 ± 0.80	0.09 ± 0.75	0.460
	6–10 mm	10.42 ± 1.36	10.47 ± 1.50	0.06 ± 0.66	0.589
	Total	10.29 ± 0.78	10.37 ± 0.96	0.08 ± 0.73	0.500
Total	0–2 mm	16.87 ± 1.11	18.39 ± 2.04	1.52 ± 2.30	<0.001 *
	2–6 mm	15.12 ± 0.76	16.21 ± 1.21	1.09 ± 1.19	<0.001 *
	6–10 mm	13.99 ± 1.98	14.07 ± 2.02	0.08 ± 0.69	0.470
	Total	15.02 ± 1.02	15.79 ± 1.39	0.77 ± 1.05	<0.001 *

CD = corneal densitometry; the difference value is expressed as postoperative minus preoperative. All values are displayed in the form of mean ± standard deviation. *: *p* values < 0.05 at *t*-test.

**Table 3:** Correlation analysis of different factors with changes in total CD of keratoconus.

Factor	0–2 mm		2–6 mm		6–10 mm		Total	
	r	*p*	r	*p*	r	*p*	r	*p*
Age	0.397	0.010 *	0.374	0.016 *	0.121	0.451	0.377	0.015 *
Kf (D)	−0.072	0.655	0.044	0.787	0.139	0.387	0.025	0.879
Ks (D)	0.056	0.730	0.199	0.212	0.231	0.147	0.176	0.272
TCT (μm)	−0.098	0.540	−0.131	0.414	−0.230	0.148	−0.163	0.308
Astig F (D)	0.243	0.126	0.361	0.021 *	0.267	0.091	0.341	0.029 *
Ecc F	0.038	0.812	0.222	0.162	0.244	0.125	0.182	0.255
Aspher F	0.012	0.939	−0.164	0.305	−0.227	0.153	−0.129	0.422
Ecc B	−0.018	0.910	0.139	0.386	0.263	0.097	0.124	0.439
Aspher B	0.016	0.921	−0.145	0.366	−0.246	0.120	−0.123	0.442
Kmax (D)	0.071	0.660	0.238	0.134	0.223	0.161	0.198	0.215
ISV	−0.060	0.711	0.122	0.446	0.166	0.299	0.073	0.650
IVA	−0.082	0.608	0.067	0.678	0.110	0.492	0.023	0.886
I-S	−0.101	0.531	−0.007	0.964	0.011	0.943	−0.045	0.782
KISA%	−0.014	0.932	0.104	0.518	−0.085	0.595	0.019	0.906

Aspher B = back corneal asphericity; Aspher F = front corneal asphericity; Astig F = front corneal astigmatism; Ecc B = back corneal numeric eccentricity; Ecc F = front corneal numeric eccentricity; ISV = index of surface variance; IVA = index of vertical asymmetry; Kf = flat keratometry; Ks = steep keratometry; TCT = thinnest corneal thickness. Kmax refers to maximum anterior sagittal curvature; I-S refers to difference in refractive power between inferior and superior at 6 mm diameter of cornea; KISA% refers to values based on a combination of keratometry, I-S, skewed radial axis, and astigmatism in Pentacam. *: *p* values < 0.05 at correlation analysis.

## Data Availability

The datasets generated and analysed during the current study are not publicly available. However, they are available from the corresponding author on reasonable request.

## Supplementary Materials

The following supporting information can be downloaded at https://www.mdpi.com/article/10.3390/jcm15083137/s1, Table S1: Correlation analysis between different factors (baseline values) and ΔCD in different areas of cornea before and after accelerated CXL in keratoconus.

## Abbreviations

The following abbreviations are used in this manuscript:

KC	Keratoconus
CXL	Corneal collagen crosslinking
CD	Corneal densitometry
D	Dioptre
TCT	Thinnest corneal thickness
GSU	Grey scale units
tCD	Total corneal densitometry
UV	Ultraviolet
BCVA	Best corrected visual acuity
