# Supplementary material for: Changes in Corneal Densitometry and Relationship to Corneal Topographical Parameters in Accelerated Corneal Crosslinking for the Treatment of Keratoconus
Source: J Clin Med. 2026 Apr 20;15(8):3137. doi: 10.3390/jcm15083137 (PMC13118013; doi:10.3390/jcm15083137)
Supplement: Supplementary file 1 [file jcm-4180805-supplementary.pdf › jcm-4180805-supplementary.pdf]

Supplementary Table S1 Correlation analysis between different factors (baseline values) and ΔCD in different areas of cornea before and after accelerated CXL in keratoconus

	Anterior layer								Center layer								Posterior layer							
	0-2mm		2-6mm		6-10mm		Total		0-2mm		2-6mm		6-10mm		Total		0-2mm		2-6mm		6-10mm		Total	
	r	P	r	P	r	P	r	P	r	P	r	P	r	P	r	P	r	P	r	P	r	P	r	P
Age	0.384	0.013*	0.336	0.032*	0.137	0.392	0.359	0.021*	0.316	0.044*	0.406	0.008*	0.063	0.694	0.347	0.026*	0.267	0.091	0.148	0.355	0.060	0.707	0.165	0.302
Kf (D)	-0.023	0.887	0.123	0.445	-0.016	0.922	0.046	0.774	-0.231	0.147	-0.128	0.424	-0.026	0.873	-0.175	0.273	-0.312	0.047*	0.004	0.980	-0.130	0.417	-0.142	0.377
Ks (D)	-0.059	0.712	0.061	0.707	-0.041	0.801	-0.005	0.976	-0.163	0.309	-0.189	0.236	0.030	0.853	-0.150	0.350	-0.268	0.091	0.065	0.688	-0.024	0.881	-0.065	0.687
TCT (μm)	-0.094	0.541	-0.386	0.013*	-0.214	0.179	-0.047	0.703	-0.179	0.171	-0.083	0.591	-0.229	0.150	-0.053	0.431	-0.190	0.181	-0.091	0.441	-0.367	0.018*	-0.116	0.494
Astig F	-0.054	0.735	-0.024	0.882	-0.048	0.768	-0.445	0.004*	0.006	0.972	-0.147	0.360	0.076	0.639	-0.035	0.830	-0.080	0.620	0.083	0.607	0.093	0.563	0.443	0.005*
Ecc F	-0.148	0.357	0.062	0.699	0.390	0.012*	0.045	0.778	-0.169	0.290	-0.007	0.964	0.304	0.053	0.014	0.931	-0.076	0.636	0.106	0.508	0.180	0.261	0.085	0.599
Aspher F	0.187	0.241	-0.052	0.749	-0.215	0.177	0.013	0.936	0.278	0.078	0.140	0.384	-0.215	0.178	0.125	0.437	0.265	0.094	0.002	0.989	-0.060	0.711	0.061	0.703
KMax (D)	-0.155	0.203	-0.387	0.012*	-0.245	0.122	-0.237	0.094	-0.397	0.014*	-0.063	0.472	-0.122	0.447	-0.127	0.441	-0.099	0.471	-0.234	0.142	-0.176	0.270	-0.314	0.046*
Ecc B	0.265	0.094	0.354	0.023*	0.331	0.035*	0.155	0.243	0.278	0.098	0.051	0.524	0.283	0.073	0.193	0.251	0.088	0.584	0.250	0.114	0.119	0.458	0.173	0.280
Aspher B	-0.029	0.836	-0.024	0.845	-0.106	0.508	-0.132	0.435	-0.076	0.615	-0.107	0.530	-0.410	0.008*	-0.090	0.501	-0.180	0.260	-0.163	0.320	-0.242	0.127	-0.292	0.064
ISV	-0.136	0.294	-0.104	0.339	-0.076	0.636	-0.102	0.352	-0.141	0.324	-0.245	0.123	-0.029	0.857	-0.328	0.036*	-0.279	0.078	-0.315	0.045*	-0.138	0.391	-0.265	0.094
IVA	-0.142	0.249	-0.361	0.020*	-0.050	0.754	-0.135	0.332	-0.337	0.031*	-0.174	0.277	-0.026	0.874	-0.246	0.121	-0.162	0.311	-0.274	0.083	-0.095	0.556	-0.197	0.218
I-S	-0.074	0.616	-0.197	0.510	-0.058	0.719	-0.168	0.218	-0.326	0.038*	-0.208	0.191	-0.029	0.858	-0.257	0.105	-0.174	0.277	-0.318	0.043*	-0.111	0.489	-0.224	0.159
KISA%	-0.144	0.368	-0.200	0.211	0.057	0.722	-0.148	0.355	-0.039	0.808	0.019	0.908	0.194	0.225	0.052	0.746	0.001	0.993	0.028	0.864	0.163	0.310	0.070	0.663

Aspher B = back corneal asphericity; Aspher F = front corneal Asphericity; Astig F = front corneal astigmatism; CD = corneal densitometry; Ecc B = back corneal numeric eccentricity; Ecc F = front corneal numeric eccentricity; ISV = index of surface variance; IVA = index of vertical asymmetry; Kf = flat keratometry; Ks = steep keratometry; TCT = thinnest corneal thickness. Kmax refers to maximum anterior sagittal curvature; I-S refers to difference in refractive power between inferior and superior at 6 mm diameter of cornea; KISA% refers to values based on a combination of keratometry, I-S, skewed radial axis, and astigmatism in Pentacam. *: P values < 0.05 at correlation analysis.
